# WormTagDB: a systematic survey of endogenously tagged proteins in *Caenorhabditis elegans* and roadmap toward the tagged proteome

**DOI:** 10.1093/g3journal/jkag068

**Published:** 2026-03-24

**Authors:** Jake Leyhr, Qiuyi Chi, Lin Zeng, Xuejia Li, Beibei Cao, En-Zhi Shen, Wei Zou, David R Sherwood

**Affiliations:** Department of Biology, Duke University, 130 Science Drive, Durham, NC 27708, United States; Department of Biology, Duke University, 130 Science Drive, Durham, NC 27708, United States; School of Life Sciences, Westlake University, Hangzhou, Zhejiang 310024, China; School of Life Sciences, Westlake University, Hangzhou, Zhejiang 310024, China; Institute of Translational Medicine, Zhejiang University, Hangzhou, Zhejiang 310027, China; School of Life Sciences, Westlake University, Hangzhou, Zhejiang 310024, China; Institute of Translational Medicine, Zhejiang University, Hangzhou, Zhejiang 310027, China; Department of Biology, Duke University, 130 Science Drive, Durham, NC 27708, United States

**Keywords:** endogenous tagging, knock-in allele, *C. elegans*, CRISPR-Cas9, genome editing, fluorescent proteins, epitopes, primer design

## Abstract

Endogenous protein tagging in *Caenorhabditis elegans* enables the direct visualization and manipulation of proteins in vivo, providing native readouts of expression, localization, and dynamics. No coordinated effort currently exists to comprehensively tag proteins on a large scale, resulting in patchy coverage that limits proteome-wide analyses. We systematically reviewed 2,500 primary research articles, identifying 778 that report novel endogenous tags, and integrated these with the Caenorhabditis Genetics Center strain records to catalog >90% of all existing tagged alleles. In total, we found that 1,554 unique genes (∼8% of the proteome) have been endogenously tagged. Gene Ontology enrichment analysis revealed that cytoskeletal proteins, transcription factors, and RNA-binding proteins dominate the tagged proteome, while membrane proteins, metabolic enzymes, and mitochondrial components remain largely untagged, reflecting both technical barriers and research priorities that have shaped the last decade of tagging efforts. We created WormTagDB (https://wormtagdb.rc.duke.edu), an interactive, community-updatable resource that consolidates all known endogenously tagged alleles and provides precomputed CRISPR guide and homology-arm primer designs for N- and C-terminal knock-ins across all protein-coding genes. This will enable researchers to easily identify existing alleles to prevent redundant strain generation and rapidly initiate new knock-in experiments. A systematic effort to tag every *C. elegans* gene would deliver a complete metazoan visual proteome, providing comprehensive insights into protein localization, dynamics, and regulation, revealing new protein associations and molecular processes.

## Teaser

Endogenous protein tagging allows researchers to visualize protein localization and function in living *C. elegans* worms, but no comprehensive catalog of tagged alleles exists. Leyhr *et al*. manually surveyed 2,500 research papers and CGC records, identifying 1,554 tagged genes (~8% of the proteome). They created WormTagDB, a centralized community-updatable database that consolidates all known tagged alleles and provides precomputed CRISPR reagent and primer designs for every protein-coding gene. This will prevent redundant strain generation and streamline time-consuming reagent design across the community. Their analysis reveals biases in the current tagging landscape and establishes a roadmap for tagging the whole proteome.

## Introduction

The insertion of fluorescent, epitope, and affinity tags directly into native gene loci, known as endogenous tagging, has become a foundational tool in modern cell and developmental biology (Leonetti et al. 2016; Kanca et al. 2017). In this approach, the tag sequence is fused in-frame with the coding region of the target gene, allowing the resulting fusion protein to be expressed under native regulatory control and translated as a contiguous polypeptide. By preserving endogenous expression levels and regulatory contexts, this approach allows researchers to conduct biochemical and microscopy studies of protein dynamics, localization, and interactions in vivo without the confounding effects of overexpression or ectopic promoters (Frøkjær-Jensen et al. 2008; Nance and Frøkjær-Jensen 2019). Endogenously tagged alleles now underpin a wide array of imaging, biochemical, and functional studies across model organisms (Huh et al. 2003; Keeley et al. 2020; Cho et al. 2022; Xu et al. 2022).

Over the last 3 decades, techniques have evolved to achieve efficient endogenous tagging in *Caenorhabditis elegans*. Early attempts at gene editing via homologous recombination proved technically challenging with a low frequency of edits (e.g. Broverman et al. 1993), limiting practical utility. A major advance came in the early 2000s with the development of transposon-based recombination methods. Using excision of a Tc1 transposon inserted in the target gene to stimulate homologous recombination, Barrett et al. (2004) published the first fluorescently tagged protein expressed from its native locus in the worm by inserting GFP into the C-terminus of the *frm-3* gene. While still technically challenging and inefficient, this work demonstrated that precise tagging at endogenous loci was feasible. Building on the principle of transposon-stimulated editing, the MosTIC system introduced a more generalizable method using engineered Mos1 elements (Robert and Bessereau 2007). This approach enabled targeted excision of a Mos1 insertion followed by homology-directed repair to insert GFP into the native *unc-5* locus. The development of CRISPR-Cas9 genome editing in 2013 marked a turning point in endogenous fluorophore tagging in the worm (e.g. Dickinson et al. 2013). Cas9-directed double-strand breaks, coupled with homology-directed repair, allowed precise tag insertion at endogenous loci. The development of user-friendly tools, including the self-excising cassette (SEC) system (Dickinson et al. 2015), co-CRISPR screening (Arribere et al. 2014; El Mouridi et al. 2017), and cloning-free protocols (Paix et al. 2015), increased efficiency and led to the generation of tagged alleles becoming a routine practice. Recently, as the community's ambitions have grown toward tagging the proteome at scale and with greater tag diversity, novel modular (Han et al. 2026; Hefel et al. 2026) and high-throughput (Eroglu and Hobert 2026) methods have emerged.

Despite the widespread adoption of endogenous tagging in *C. elegans*, there is not yet a centralized, searchable inventory of labeled genes. WormBase and the Caenorhabditis Genetics Center (CGC) serve as vital resources for cataloging alleles and strains, but their coverage of endogenously tagged alleles is incomplete, with many only documented in the text or supplementary information of individual research articles. This fragmentation hinders both experimental planning and community-wide coordination. Researchers can also duplicate existing alleles unknowingly, missing the opportunity to share already validated alleles that could accelerate studies. For example, Riga et al. (2021) C-terminally tagged *hmr-1* with GFP years after Marston et al. (2016) published an identical allele, while Nguyen and Phillips (2021) and Charlesworth et al. (2021) independently and near-simultaneously generated functionally identical FLAG::GFP::*alg-3* alleles.

Here, we performed a manual survey of the *C. elegans* literature, screening 2,500 papers to identify ∼90% of published alleles in which a fluorescent or other protein tag was inserted into a gene's endogenous locus. We integrated this dataset with CGC strain records to generate a comprehensive inventory of endogenously tagged genes in *C. elegans*. We developed WormTagDB (https://wormtagdb.rc.duke.edu), an interactive RShiny platform, to make this resource publicly accessible, searchable, and community-updatable. We also analyzed Gene Ontology (GO) term enrichment to uncover systematic trends in the current tagged gene set. Based on these findings, we propose a roadmap for a coordinated, community-driven effort to more rapidly advance the tagging of the *C. elegans* proteome.

## Methods

To identify endogenously tagged *C. elegans* alleles described in the literature, a keyword-based search was performed using WormBase's Textpresso Central portal (https://wb-textpresso.alliancegenome.org/tpc/search; Müller et al. 2018), updated as of September 1, 2025. The search included the following terms commonly associated with endogenous genome editing: “knock-in,” “knock in,” “tagged,” “CRISPR,” “gRNA,” “homology arm,” and “repair template.” This search returned 11,015 papers, each assigned a keyword relevance score by the Textpresso engine. The top 2,500 papers were manually reviewed in descending order of score, including both main text and supplementary materials. Of these, 778 were found to describe the generation of 1 or more novel endogenously tagged alleles. For each paper, all newly generated alleles representing the insertion of protein tags at native genomic loci were recorded. Endogenously tagged strains obtained from other sources, such as previously published studies or external collaborators, were excluded unless the paper described their de novo generation. In cases where multiple functionally identical alleles were reported for the same tagged gene (e.g. independent insertions of *gene-1::GFP* from separate microinjections or founders), only 1 representative allele was recorded to avoid redundancy. In rare cases, the generation of specific alleles was described in more than 1 publication, and in these cases, only the earliest publication was kept. Constructs introduced via MosSCI, multicopy transgenes, or overexpression arrays were excluded from the analysis. Also excluded were self-cleaving transcriptional reporters (e.g. T2A and SL2) and endogenously tagged mutant loci. This literature-curated allele list was merged with the set of endogenously tagged alleles available through the CGC, based on CGC strain records as of September 1, 2025. The final dataset represents a manually curated, up-to-date inventory of endogenously tagged *C. elegans* alleles as of this date.

Segmented regression analysis of annual rates of newly tagged genes was performed using the *segmented* R package (v2.1-4) (Muggeo 2008). GO annotations for *C. elegans* protein-coding genes were obtained from Ensembl BioMart and evidence codes from org.Ce.eg.db (v3.20.0) (Carlson 2024). GO term similarity and hierarchical clustering were performed using the rrvgo (v1.18.0) Bioconductor package (Sayols 2023), with semantic similarity calculated via the Wang method and a similarity threshold of 0.7 for clustering related biological process (BP) and molecular function (MF) terms and 0.6 for Cellular Compartment (CC) terms. The GO terms in each cluster with the largest count of Entrez Gene identifiers annotated to that GO term or to its child nodes in the ontology were selected as the parent term for the cluster. Enrichment was assessed by calculating the proportion of tagged genes per GO term and comparing it to the proportion expected if genes were tagged at random across the genome. GO terms were grouped by parent category to reveal higher-order functional trends in tagging coverage. Disease ontology (DO) annotations were obtained from Alliance of Genome Resources v8.1.0 (https://www.alliancegenome.org/downloads). The number of tagged genes in each GO term or disease category was compared to the total number of *C. elegans* genes associated with that GO term/disease to calculate an odds ratio, which was log2 transformed for visualization. Statistical significance was determined using Fisher's exact test with a false discovery rate (FDR) threshold of 0.05. For visualization, a log2(odds ratio) cutoff of 7 and −7 was used in place of infinite values to allow GO terms/diseases with completely tagged or untagged gene sets to be plotted on the same scale.

To make the curated dataset accessible to the broader research community, we developed the interactive web application WormTagDB using the RShiny framework. The app allows users to browse, search, and filter all identified endogenously tagged alleles by gene, tag type, tag position, GO terms, and human disease associations. Each entry includes the gene name, tag, allele designation, source, and strain information where available. Users can also download the complete dataset and submit new alleles through an integrated community submission form. The app is publicly available at https://wormtagdb.rc.duke.edu and will be updated regularly to reflect newly published alleles. The code is available at https://github.com/jakeleyhr/WormTagDB.

To design genome-wide N- and C-terminal tagging sites and corresponding CRISPR guides, we created custom Python scripts (https://github.com/jakeleyhr/CRISPR-Guide-and-Primer-Design-Pipeline-for-C.-elegans). We began with a list of all protein-coding *C. elegans* genes, selected the Ensembl canonical transcript and corresponding UniProt ID, and inferred mature protein boundaries in a “chain-aware” manner by parsing curated features (initiator methionine removal, signal peptides, propeptides, transit peptides, lipidation sites, and annotated chain regions). The N-terminal insertion site was positioned immediately downstream of all predicted N-terminal processing events (initiator-Met removal and any signal, transit, or propeptide segments), and the C-terminal site was positioned immediately upstream of residues expected to be removed. Insertion sites were positioned 5aa proximal to any terminal lipidation sites. Simple motif searches for PTS1, PTS2, KDEL, di-lysine, and CAAX motifs were also performed, but as these were of lower confidence, they did not influence insertion site choice and instead were listed as potential warning flags. N- and C-terminal insertion coordinates were defined at the nucleotide immediately 5′ of the relevant codon boundary in transcript orientation by converting amino acid indices to coding sequence (CDS) positions (WBcel235). For each site, ±50 bp of genomic sequence was retrieved, and both strands were scanned for SpCas9 NGG targets, evaluating candidates as 20-bp protospacer + NGG PAM with cut positions at the expected offset 3 bp upstream of the protospacer adjacent motif (PAM) site. Candidates were filtered for composition: GC between 25% and 80%, no 7+-bp-long homopolymers, and divided into guides suitable for “in vivo transcription” or “in vitro transcription only” based on the absence/presence of 4+ consecutive Ts. Candidates with GGG PAM sites or NGG PAM sites where the next nonguide base was G (NGG + G) were also excluded. Potential off-target cut sites were identified using FlashFry (McKenna and Shendure 2018). Guides were ranked by the minimal number of off-target sequences and then by the proximity of the cut site to the intended insertion coordinate. The best single “in vivo transcription” guide was selected for each target site, with an additional “in vitro transcription only” guide reported if it had a cut site closer to the insertion site.

For each N- or C-terminal target locus, we designed primers to amplify the left (primer 1 and primer 2) and right (primer 3 and primer 4) homology arms used to flank the knock-in cassette. We first extracted ±100 bp of genomic sequence surrounding the predicted insertion site, and primers 2 and 3 were directly anchored to the genomic sequence immediately adjacent to the insertion site: Primer 2 comprised the 30 to 35 bp immediately upstream, and Primer 3 the 30 to 35 bp immediately downstream. Four to five silent mutations were introduced at codons in the primers corresponding to the 3′ end of the guide and PAM site to prevent cutting of the vector or repaired allele. When a guide cut site was located far enough from the insertion site that not all required silent mutations could be placed within Primers 2 or 3 while still retaining at least 15 unmodified bases at the 3′ end, an overlapping 2-primer design was used. In these cases, auxiliary primers (primers 2A or 3A) containing the necessary silent mutations were generated with ∼20-bp overlaps to the modified primers 2 or 3 (renamed primers 2B or 3B).

Primers 1 and 4 were then identified using the Primer3 program (Untergasser et al. 2012) to pair appropriately with primers 2 and 3, respectively, with the goal of generating homology arm amplicons between ∼500 and 1000 bp and with closely matched annealing temperatures. If no valid pairing was found within the default search window, the maximum allowable arm length was incrementally expanded up to 10 kb. All candidate primer 1 and 4 sequences were subjected to BLAT searches against the *C. elegans* genome, and when possible, only primers without predicted off-target matches were selected. Finally, genotyping primers flanking the entire edited region were designed using Primer3, again using BLAT searches on candidates to ensure high specificity. These primers can also be used in a 2-step nested workflow in which the locus is first amplified by the genotyping PCR primers, followed by a second PCR reaction to obtain each homology arm.

## Results and discussion

### Literature survey of endogenously tagged genes

To systematically identify *C. elegans* endogenously tagged genes, we performed a keyword-based search using WormBase's Textpresso platform. Search terms were chosen to capture studies employing genome editing for native locus tagging, including “knock-in,” “knock in,” “tagged,” “CRISPR,” “gRNA,” “homology arm,” and “repair template.” This search returned 11,015 publications, each scored and ranked by keyword relevance. We manually reviewed the top 2,500 papers. This number was chosen as discovery curve analysis with an exponential saturation model indicated that ∼90% gene coverage would be achieved by examining ∼2,424 papers (Supplementary Fig. 1). Within this group of papers, 778 reported the generation of 1 or more new endogenously tagged alleles (Supplementary Table 1). Each allele was annotated for gene identity, tag type(s), tag location, and additional metadata when available. This dataset was then combined with the list of endogenously tagged strains cataloged by the CGC to generate the final endogenously tagged database (Supplementary Table 2).

**Fig. 1.**
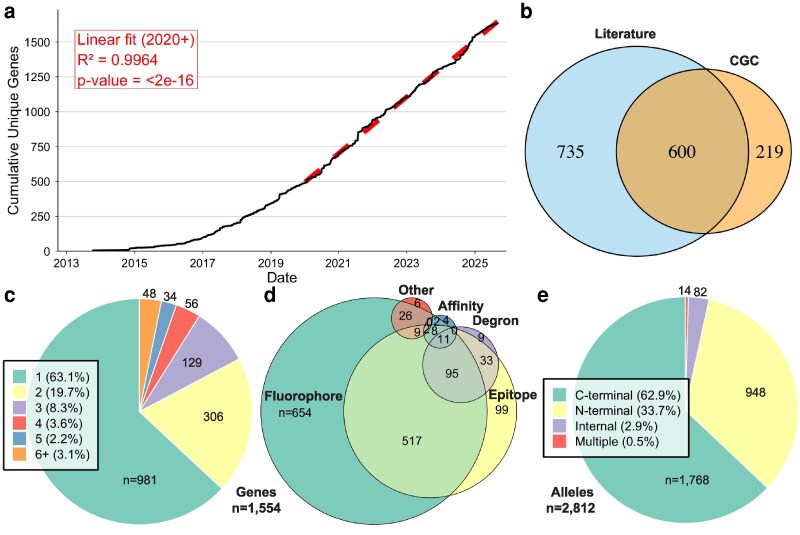
Landscape of endogenously tagged genes in *C. elegans*. a) Cumulative number of unique genes tagged endogenously over time. Tagging has increased linearly since 2020, with an average rate of ∼203 new genes per year (linear fit: R^2^ = 0.9964, *P* < 2e-16). b) Overlap between genes tagged in the published literature and those available from the CGC. c) Number of distinct alleles per gene. Most genes (63.1%) have only 1 reported tagged allele, while a minority have multiple independently generated alleles. d) Distribution of tag types across all genes. Many genes are tagged with multiple tag classes, including fluorescent proteins, epitopes, degrons, affinity tags, and others. e) Tag insertion positions across all alleles. C-terminal tagging is most common (62.9%), followed by N-terminal (33.7%) and internal insertions (2.9%). A small number of alleles (0.5%) carry multiple tags at different positions.

Figure 1 provides an overview of the compiled dataset, including the number of newly tagged genes reported since 2013, the distribution of tag types, and the relative proportions of tag placements. In total, we identified 2,812 different alleles, which tagged 1,554 unique genes. By examining the genes endogenously tagged over time, we found that tagging activity expanded dramatically following the introduction of CRISPR–Cas9 genome editing in 2013 (Fig. 1a). However, since 2020, the number of unique genes tagged per year has held steady at ∼200 new genes per year rather than increasing (Supplementary Fig. 2).

Given that researchers usually contribute their most significant published strains to the CGC, we expected, and found, a considerable overlap between tagged genes described in the literature and those found in the CGC database (Fig. 1b). However, 47% of all tagged genes are not available from the CGC. In many cases, genes have been tagged in multiple ways by different labs, so there are more generated alleles than tagged genes. Of all the published alleles, 92% had allele designations (e.g. qy108), which were used to cross-reference with the CGC database. This revealed that most alleles (70%) have not been deposited with the CGC and that 42% of CGC alleles are not yet described in the literature, although this latter number may decrease with a more exhaustive literature survey. We next examined the proportions of genes that had multiple tagged alleles. Most genes (63.1%) were represented by a single allele, 19.7% by 2, 8.3% by 3, while the remaining 8.9% have been tagged in 4 or more alleles (Fig. 1c). In some cases, these reflect different tags or insertion sites designed for specific applications (e.g. dual-color imaging or biochemical assays). In others, they represent redundant alleles generated independently by different labs (e.g. 177 genes have 2 or more alleles tagged with similar fluorophores by different labs).

The most frequently used tags were fluorophores and epitopes (Fig. 1d). Among these, GFP (44.0% of alleles), mNeonGreen (17.0%), and FLAG tags (30.1%) were most common, with the majority placed at the C-terminal end of the protein (62.9%), followed by the N-terminus (33.7%), while only a small minority (2.9%) were internally inserted (Fig. 1e). This bias toward the C-terminus likely reflects the biological and practical advantages of placing tags at the protein's C-terminus, such as reduced chance of disruption of regulatory elements near the transcriptional start site, and that N-terminal SEC insertions can be challenging to create as they behave as loss-of-function alleles prior to excision (Dickinson et al. 2015).

Taken together, these observations reveal that to date, without a centralized effort, ∼8% of the *C. elegans* proteome has been tagged. In addition, more than 10% of these proteins have been redundantly labeled. These results indicate the feasibility and progress toward a complete proteome, but also underscore the need for a centralized tracking system and communication of available strains between researchers for greater efficiency.

### The current tagging landscape

**Fig. 2.**
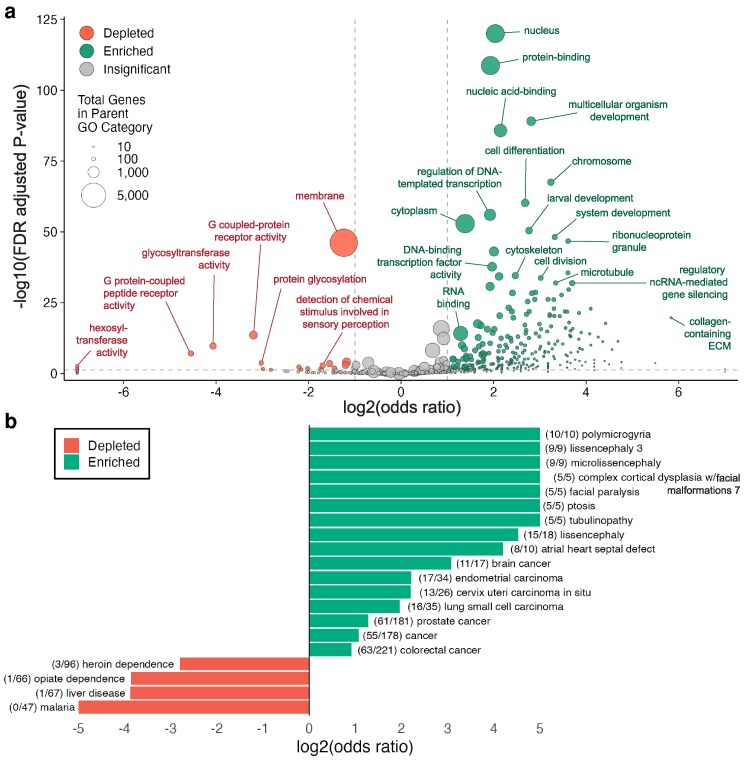
Functional and disease enrichment patterns reveal selective biases in endogenous tagging efforts. a) Volcano plot showing GO term enrichment analysis. Each point represents a GO parent category following clustering by semantic similarity (728 total categories). b) Human disease-associated gene enrichment analysis showing log2(odds ratio) for disease categories significantly enriched or depleted among tagged genes. Numbers in parentheses indicate (tagged genes/total genes) in each disease category.

To better understand how existing tagging efforts have been distributed across the *C. elegans* genome, we compared the set of 1,554 tagged genes to the genome-wide background of 14,956 protein-coding genes annotated with at least 1 GO term. Overall, 10.0% of genes with GO terms have been tagged (1,495 genes), compared with 7.8% of all genes. To identify representation within broader GO categories, GO terms were clustered by semantic similarity, reducing 6,800 individual terms into 728 broader parent categories (414 BPs, 197 MFs, and 117 CCs) that consolidate related functions and gene sets. We then performed GO term enrichment analysis to determine if there was over- or underrepresentation of tagged genes among functional categories (Fig. 2a). Relatively few functional categories were underrepresented for endogenously tagged alleles, whereas many categories showed strong enrichment.

The depleted set of parent terms skews toward membrane-associated and enzyme-heavy categories. Broad membrane terms are underrepresented (membrane 390/6508 tagged, 6.0%), with low coverage in organelle interiors (mitochondrial matrix 7/177, 4.0%) and G-protein-coupled receptor (GPCR) functions (GPCR activity 6/484, 1.2%; peptide GPCR 1/208, 0.48%). Glycosylation and redox pathways are similarly underrepresented (glycosyltransferase activity 2/297, 0.7%; hexosyltransferase activity 0/75, 0%; oxidoreductase activity 27/497, 5.4%). This pattern is consistent with the difficulty of tagging multipass transmembrane and organelle-targeted proteins without disrupting folding, trafficking, import, or activity (Harner et al. 2011; Soave et al. 2021).

The enriched GO categories among endogenously tagged genes are likely due to a convergence of scientific interest and where fluorescent protein tagging has been technically feasible. Cytoskeletal and chromosome-linked components are highly represented (microtubule cytoskeleton 69/133, 51.9%; chromosome 154/314, 49.0%), in line with longstanding interest in spindle assembly, division, and morphogenesis (e.g. Honda et al. 2017; Nishida et al. 2021; Xu et al. 2024). Collagen-containing extracellular matrix also stands out (25/29, 86.2%), consistent with extensive studies of cuticular collagens, basement-membrane dynamics, and tissue remodeling (Keeley et al. 2020; Jayadev et al. 2022; Adams et al. 2023; Ragle et al. 2025). Broader enriched categories include RNA regulation (ribonucleoprotein granule 92/164, 56.0%; regulatory ncRNA-mediated gene silencing 62/217, 28.6%), protein–protein and protein–DNA interactions (protein binding 641/2856, 22.4%; nucleic acid binding 372/1308, 28.4%, DNA-binding transcription factor activity 177/624, 28.4%), and developmental biology terms including cell differentiation (177/453, 39.1%), multicellular development (248/620, 40.0%), larval development (141/345, 40.9%), and neurogenesis (57/107, 54.3%).

Human disease-associated genes are also enriched, with 18.0% tagged (813/4517) compared to 7.8% of all genes. Enrichment analysis of specific disease categories revealed strong overrepresentation of tagged genes in several rare disease groups, including tubulinopathy, polymicrogyria, ptosis, facial paralysis, and multiple lissencephaly subtypes (Fig. 2b). This is a direct result of these diseases being driven by defects in microtubule biology, which is a well-tagged protein class. Atrial heart septal defect is likewise enriched as myosins (*unc-54, myo-2*) and homologs of transcriptional regulators involved in heart development (e.g. *ceh-28*, *elt-1*) have been well tagged. Finally, multiple cancers, some of which encompassed larger gene sets (e.g. colorectal and prostate cancer), appear due to their overlap with broad enriched classes, including transcriptional and cell-cycle regulators. On the other hand, while many diseases have very few or zero tagged genes, only 4 categories—heroin dependence, opiate dependence, liver disease, and *Plasmodium falciparum* malaria—were significantly depleted for tagged genes. The genes associated with these diseases are strongly enriched for metabolic and detoxification GO terms, including xenobiotic metabolism, monooxygenase and oxidoreductase activity, and heme/iron binding, reflecting that components in these relatively large pathways remain almost entirely untagged in *C. elegans*.

## Best practices and tagging considerations

An important element for accelerating tagging of the *C. elegans* proteome is establishing a pipeline to guide researchers in designing, generating, validating, and sharing endogenously tagged alleles (Dickinson et al. 2015; Nance and Frøkjær-Jensen 2019). Below, we concisely review the best practices for each step of the process, from choosing protein tags and their insertion locations to allele validation.

### Choosing the insertion location

A critical first step in all protein tagging experiments is a detailed analysis of the protein's structure, functional domains, and posttranslational modifications. Tags inserted near enzymatically active sites, binding motifs, regulatory domains, or transmembrane segments can impair protein activity, stability, or localization. Likewise, motifs such as nuclear localization or export signals, signal peptides, residues subject to N-terminal processing (e.g. initiator methionine removal or myristoylation, signal peptide cleavage), and internal proteolytic cleavage sites must be carefully considered, as tagging in these regions can disrupt essential functions or result in the tag being removed during processing (Tian et al. 2004; Huang et al. 2010). Importantly, “N-terminal tagging” does not always place the tag at the initiating methionine. For proteins with a cleaved N-terminal signal peptide, the tag should be positioned immediately downstream of the cleavage site, effectively generating an N-terminally tagged mature protein while preserving signal peptide function (Keeley et al. 2020). Internal tagging is also a viable strategy when the region of interest is structurally permissive and shared across isoforms. This can involve inserting the tag into an internal exon, or insertion via a synthetic exon that includes minimal splice sites (e.g. 5′ CAG and 3′ GTA; Lim and Burge 2001) engineered into an intron of the target gene (Keeley et al. 2020). Bioinformatic tools and databases such as AlphaFold, UniProt, and Pfam provide valuable structural and domain annotations, including catalytic cores, domain boundaries, signal peptides, and intrinsically disordered regions. Disordered regions and surface-exposed internal loops often provide flexible and permissive sites for tag insertion. Combining these annotations with quantitative features, such as the degree of surface exposure of each amino acid position (relative solvent accessibility) and evolutionary sequence conservation (indicating functional regions), can reveal permissive tagging locations (Xu et al. 2024; Zinski et al. 2024).

The diversity of transcript isoforms presents an additional layer of complexity. Alternative transcription start sites or splicing events may alter N- or C-terminal CDSs, subcellular targeting signals, or expression profiles. In many cases, isoforms arise from alternative transcription start sites or exon skipping near the 5′ end of the gene, meaning that internal or C-terminal tagging is more likely to label all isoforms simultaneously (Ramani et al. 2011; Weinreb et al. 2025). However, this generalization does not hold universally, and the isoform structure of each gene should be carefully assessed using available gene models or transcriptomic data. When possible, tags should be inserted into exons shared by all biologically relevant isoforms or the canonical or most highly expressed isoform, unless a specific variant is the intended target.

Practical considerations during CRISPR-mediated knock-in can also influence tag placement. For example, in strategies that rely on the SEC system (Dickinson et al. 2015), N-terminal tagging involves an initial insertion that separates the promoter and target gene CDS, producing a knockout allele prior to selection cassette excision. This step can be problematic for essential genes, where loss of function is lethal or causes selection bottlenecks. In such cases, C-terminal tagging is often more feasible, as animals will carry the desired insertion without passing through a strong loss-of-function intermediate generation. However, a recently reported SEC variant (NSEC) avoids this N-terminal knockout intermediate by embedding the selection cassette in a reverse orientation within an artificial intron that is spliced out of the target gene's mRNA, preserving native gene function throughout strain construction (Gibney and Pani 2026). This innovation removes this common barrier to N-terminal endogenous tagging.

### Tag types

Once the optimal location for tag insertion has been established, tag selection is another important feature to be considered. Bright fluorescent proteins such as GFP, mNeonGreen, and mScarlet are widely used for live-cell imaging of protein localization and dynamics, but their relatively large size (∼27 kDa) can hinder proper folding, trafficking, or function, especially when tagging small, highly structured, or sterically restricted proteins such as ribosomal subunits or transcription factors (Noma et al. 2017; Costa et al. 2023). In such cases, split fluorophore systems provide a valuable alternative (Cabantous and Waldo 2006). For example, tagging with the small 1.8-kDa sfGFP(11) peptide, combined with tissue-specific expression of the complementary 24-kDa sfGFP(1-10) fragment, minimizes the size of the inserted tag while enabling spatially restricted reconstitution of fluorescence (Hefel and Smolikove 2019; Costa et al. 2023). Animal viability can also be affected by the specific fluorophore used in tagging, perhaps due to differences in fluorophore protein folding rates (Srinivasan et al. 2025).

For high-resolution or multiplexed imaging, self-labeling enzyme tags such as HaloTag (Los et al. 2008) and SNAP-tag (Keppler et al. 2004) enable covalent attachment of synthetic dyes with increased brightness, photostability, spectral range, and labeling speed compared to genetically encoded fluorescent proteins (Zhang et al. 2023a; De Luis et al. 2025). These tags offer exceptional flexibility by allowing the user to choose from a wide array of small-molecule ligands conjugated to custom fluorophores, making them especially valuable for applications requiring high signal-to-noise and optical precision, including super-resolution microscopy and single-molecule tracking (Kompa et al. 2023; Catapano et al. 2025), time-lapse imaging, and pulse-chase experiments (Borchers et al. 2025). These self-labeling tags can also be paired with specialized dyes to enable FRET-based detection of protein interactions or conformational changes, as well as the sensing of local pH, redox state, or ion concentrations (Cook et al. 2023). These capabilities make HaloTag and SNAP-tag highly versatile tools for precisely describing dynamic protein behaviors.

Smaller epitope tags, such as FLAG, HA, and V5, are widely used for biochemical applications, including Western blotting, immunoprecipitation (IP), chromatin immunoprecipitation (ChIP), and affinity purification, and also fluorescence immunostaining (Terpe 2003; Dickinson et al. 2015; De Luis et al. 2025). These tags consist of short amino acid sequences (typically 5 to 30 residues) that are recognized by specific antibodies. Their compact size minimizes (but does not eliminate, e.g. Barker et al. 2025) the likelihood of interfering with protein folding, localization, or function, even when multiple copies are inserted in tandem to increase detection sensitivity (Stowers 2025). Having multiple distinct epitopes available, each recognized by highly specific antibodies, is advantageous for multiplexed experiments in which different proteins are tagged and detected simultaneously, or for tandem immunoaffinity purification (DeCaprio and Kohl 2019; De Luis et al. 2025).

In addition to tagging endogenous proteins for visualization or biochemical studies, many strategies combine endogenous tagging of proteins with conditional control elements to enable temporal or spatial manipulation of the native protein. One widely used approach is the auxin-inducible degron (AID) system, which allows tissue-specific and temporally-controlled protein depletion by tagging the protein of interest with a minimal degron sequence and coexpressing the plant-derived TIR1 F-box protein (Zhang et al. 2015; Ashley et al. 2021). Upon auxin treatment, TIR1 recruits the SCF E3 ligase complex, leading to ubiquitination and proteasomal degradation of the degron-tagged target. A conceptually similar strategy that requires no exogenous small molecule treatment is the ZIF-1/ZF1 system, in which a short ZF1 degron tag is recognized by the endogenous ZIF-1 adaptor protein, which likewise recruits an E3 ligase complex (Armenti et al. 2014). Nanobodies can also be used to degrade proteins tagged with epitopes, such as a GFP-nanobody fused to ZIF-1 that degrades GFP-tagged proteins (Wang et al. 2017) and NbALFA fused to E3 ubiquitin ligases that degrades ALFA-tagged proteins (Yang et al. 2025). Although not formally a protein tag, knock-in protease-cleavable sites in which an internal site for a sequence-specific protease (e.g. 7aa TEV protease site) is engineered into the protein allow for inducible cleavage and inactivation upon protease expression (Harder et al. 2008 ; Das et al. 2024). Conditional expression of tagged proteins from the native locus can also be achieved using FRT-based recombinase systems, in which FLP is used to fuse a protein tag in a tissue-specific or inducible manner (Muñoz-Jiménez et al. 2017).

A common strategy that maximizes the utility of tagging is the incorporation of multiple functional elements within a single endogenous allele (39% of alleles in WormTagDB). For example, composite tags such as mNG::FLAG::AID can combine live imaging, biochemical affinity purification, and conditional protein depletion in the same strain (Dickinson et al. 2015; Sharma et al. 2024). This approach increases experimental flexibility and reduces the need to generate and validate separate alleles for each application, conserving time and resources. However, concatenating multiple tags can increase the repair template size, which may reduce insertion efficiency (Paix et al. 2016), and raise the risk of perturbing the native protein as discussed above.

### Linker sequences

To preserve native protein function, tags are often connected to the protein of interest via a flexible linker, typically composed of small, uncharged residues such as glycine and serine. These glycine-serine (Gly-Ser) repeats provide rotational freedom and reduce steric interference between the tag and adjacent protein domains. Common linkers include (Gly-Gly-Gly-Gly-Ser)_1-3_ motifs (i.e. GGGGS, repeated 1 to 3 times), which have been widely adopted due to their flexibility, hydrophilicity, and low immunogenicity (Chen et al. 2013). The optimal linker length depends on several factors, including the size and rigidity of the tag, the structural constraints of the target protein, and the location of the insertion site (e.g. near a folded domain vs in a disordered region). In *C. elegans*, the 9aa flexlink (Gly-Ala-Ser)_3_ motif was designed in the original SEC constructs (Dickinson et al. 2015), but recent studies have found that increasing this length to 18aa (Gly-Ala-Ser)_6_ (Keeley et al. 2020) or 30aa (Gibney et al. 2026) improves viability when tagging particular genes.

### Validation and documentation

Following strain generation, initial functional checks should confirm that the knock-in does not appear to interfere with the endogenous protein's function. Animals should be monitored for developmental, behavioral, and fertility defects by measuring growth rates of individual worms or time to plate starvation after placing a set number of staged worms on a defined amount of food (Srinivasan et al. 2025). However, this approach might not identify subtle phenotypes. An absence of signal or unexpected protein localization may also indicate problems such as disrupted tagged protein function, cleavage of the tag from the protein, disrupted splicing, high turnover of the protein, or low tagged protein levels. Western blotting can help determine cleavage of the tag, but an absence of signal can be difficult to interpret (Keeley et al. 2020; Srinivasan et al. 2025).

Equally important is comprehensive documentation of the tagging design, including the tag and linker sequences, precise genomic insertion site, tag orientation, and validation results, both positive and negative. Depositing this information in public repositories (e.g. AddGene, WormBase, CGC, and WormTagDB) maximizes reproducibility and enables other researchers to build on prior work while helping to identify cases in which specific tags or insertion sites subtly interfere with protein function.

## The endogenous tagging database—WormTagDB

To support community-wide access to the curated dataset of endogenously tagged *C. elegans* genes, we developed WormTagDB, an interactive web platform available at https://wormtagdb.rc.duke.edu. Built using the RShiny framework (Chang et al. 2025), the database enables researchers to browse, filter, and download tagged allele information through a simple interface. Each entry includes gene name, allele designation, tag type, tag position, source (literature or CGC), and publication reference (when available), thus facilitating direct communication and reagent sharing between labs.

While the CGC plays an essential role in strain preservation and distribution, it is not feasible for the CGC to archive every published strain. Thus, many strains remain housed in individual labs and may not be formally deposited or easily discovered. WormTagDB addresses this gap by consolidating tagged allele metadata from both the literature and CGC records, thus offering a centralized, up-to-date resource to facilitate direct lab-to-lab sharing of strains.

To ensure researchers can easily access all experimentally relevant details of the tagged alleles, WormTagDB allows users to search for specific genes, alleles, or tags, and to filter entries by tag type (e.g. GFP, mCherry, and FLAG), tag position (N-terminal, C-terminal, internal), and source (literature vs CGC). In addition, users can search for GO terms of interest (e.g. “basement membrane” or “autophagy”) to retrieve all tagged alleles of genes annotated with those terms. Results are displayed in an interactive table, and users can download the full or filtered databases as CSV files. To identify systematic functional trends in the current tagging landscape, WormTagDB also includes a GO term enrichment analysis page. This feature compares the functional annotations of tagged genes against a genome-wide background, highlighting BPs, MFs, and cellular components that are either over- or underrepresented among tagged loci. This analysis can help researchers identify saturated categories as well as areas where tagged alleles are still lacking.

To accelerate CRISPR-based endogenous tagging in *C. elegans*, WormTagDB provides predicted reagent designs for 99.6% of protein-coding genes: predicted N- and C-terminal insertion coordinates (WBcel235), sgRNA sequences, and primer sequences to amplify homology arms (Supplementary Fig. 3 and Table 3). Using a chain-aware pipeline that accounts for posttranslational processing (initiator Met removal, signal/transit/propeptides, lipidation sites, and curated chain boundaries in UniProt), we delineated the mature polypeptide. N-terminal insertion sites were positioned immediately downstream of all predicted N-terminal processing and lipidation sites, while C-terminal sites were positioned immediately upstream of C-terminal processing and lipidation sites. Lower-confidence sequence motif predictions (PTS1/PTS2, KDEL, di-Lys, CAAX) were reported as warning flags but do not influence site selection. We selected optimal guide sequences with the minimal number of off-target sequences and with cut sites closest to the target insertion location (Supplementary Fig. 3). We designed primers to amplify the flanking homology arms required for homology-directed repair, introducing silent mutations as needed to prevent Cas9 from cutting the repair template. An earlier version of this pipeline was used to successfully generate 167 tagged strains, demonstrating its utility (Supplementary Table 4). This comprehensive resource of the sequence reagents to tag 19,886 genes is accessible in the “Guide Predictions” page of WormTagDB with an Integrative Genomics Viewer interface to visualize the sequence locations and orientations, substantially lowering the barrier for entry to high-throughput tagging experiments.

WormTagDB also includes a community submission form to encourage direct contributions from researchers. Users can report newly generated endogenously tagged alleles along with associated metadata such as tag type, insertion site, linker sequence, and validation information. Using the same form, attempted knock-ins that were nonviable can also be reported. Submitted entries will be incorporated into the database to expand and update the resource, ensuring it reflects the most current tagging progress. To help coordinate ongoing work, the site also features a “reserve genes” form. This allows researchers to indicate alleles they plan to generate in the near future, optionally including their lab and contact information to be displayed in the database. By making planned projects more visible, this feature helps prevent unintentional duplication of effort, enables potential collaborators to connect early, and fosters a more coordinated, efficient approach toward systematically tagging the *C. elegans* proteome. Because WormTagDB is built around standardized metadata fields and community submissions, its architecture can also be readily adapted to support similar tagging databases in other model organisms.

## A roadmap for whole-proteome tagging

Since the advent of CRISPR-Cas9–mediated genome editing, the *C. elegans* community has made steady progress toward tagging the complete proteome. To date, 1,554 unique genes (∼8% of the proteome) have been tagged, with new additions appearing at a rate of 200 genes per year (Fig. 1a). However, at this pace, the complete proteome will not be tagged for approximately 100 years. A coordinated large-scale effort is thus needed to accelerate gene tagging. This should include laboratories sharing protocols, developing communication channels, tracking projects to increase efficiency, and training new research groups to increase participation. Many individual labs can then align efforts toward the common goal of a complete tagged proteome while maintaining flexibility to pursue their own scientific questions. Achieving this goal would also benefit from creative funding strategies that reward community contributions, whether through consortium grants for systematic tagging of prioritized gene sets or incentive structures that support individual labs for depositing validated tagged strains.

We propose that a strong candidate gene category for a coordinated tagging initiative is the ∼700 genes encoding extracellular matrix (ECM) and ECM-associated proteins that comprise the *C. elegans* matrisome. Of the 717 matrisome genes defined by Teuscher et al. (2019), 127 (17%) have already been tagged. Recent efforts to tag components of basement membrane, a conserved and specialized ECM, have already demonstrated the value of systematic ECM tagging, revealing unexpected trimer diversity in collagen IV (Srinivasan et al. 2025), nanoscale patterning of apical collagens (Adams et al. 2023), distinct localization patterns among core basement membrane components (Keeley et al. 2020), and dynamic remodeling of the spermatheca BM to facilitate stretching (Soh et al. 2025). ECM proteins are inherently difficult to study in cell culture because their function depends on the 3-dimensional tissue context in which they are assembled. A complete set of tagged matrisome alleles in *C. elegans* would provide a comprehensive view of the ECM and its dynamics in a living organism, with broad relevance to understanding tissue morphogenesis and ECM-related human diseases (Jayadev et al. 2022; Lennon and Sherwood 2025). Such data could be integrated with cross-species resources such as basement membraneBASE (https://bmbasedb.manchester.ac.uk), which catalogs basement membrane protein localization across animal species, to enable comparative analyses of ECM composition and organization.

To facilitate large-scale tagging efforts, we have provided predictions of appropriate N- and C-terminal knock-in sites along with proximal guide RNA sequences and homology arm primers for all *C. elegans* protein-coding genes on WormTagDB (Supplementary Table 3). This resource greatly streamlines the first step in knock-in generation—the manual design of these sequence reagents. This makes endogenous tagging more accessible to the broader *C. elegans* research community and will enable high-throughput tagging campaigns that would otherwise be more time-consuming.

The time required for microinjection can be another bottleneck in worm genome engineering. Manual injection typically requires 1 to 2 h per construct for experienced users, while a robotic microinjector described by Pan et al. (2024) reduces this time to ∼15 min. Further, improved reagent mixes (Paix et al. 2015) and ssDNA repair templates (Eroglu et al. 2023) can decrease the number of required injections, pushing efficiency even higher. With moderate uptake of robotic injection at institutions, the pace and accessibility of proteome-wide tagging could be dramatically accelerated.

For systematic proteome tagging, 2 complementary strategies are worth considering. The SEC strategy offers benefits for labs new to endogenous tagging or for projects focused on gene-by-gene characterization. In this approach, animals carrying the insertion are selected by drug resistance and roller phenotypes encoded on the cassette, which is subsequently removed by heat-shock induction (Dickinson et al. 2015; Gibney and Pani 2026). Although SEC requires ∼2 to 3 wk and multiple generations to obtain homozygous, scar-free alleles, it provides a straightforward entry point into genome editing with clear phenotypic markers, minimal PCR screening, and a robust, well-documented workflow. On the other hand, for large-scale tagging experiments, injections of ribonucleoprotein complexes with short linear repair templates are increasingly attractive. Paix et al. (2015) noted that 2 target loci could be edited simultaneously (in addition to the dpy-10 co-CRISPR marker locus). Recent improvements in knock-in efficiency (Eroglu et al. 2023) have made this approach more reliable and scalable, enabling the simultaneous injection of multiple repair templates to routinely tag 3 genes in a single injection round (Eroglu and Hobert 2026). This pooled approach dramatically increases throughput and reduces per-gene costs and labor.

Another important element is to standardize shared donor backbones and protein tags. As many SEC donor backbones are readily available from Addgene (e.g. Dickinson et al. 2015; Gibney and Pani 2026), backbone design is already largely standardized across the community. A remaining source of variability is fluorophore tag choice. To enable quantitative, cross-laboratory comparisons with shared strains, the community should converge on a core set (1 to 2 per color) of standardized fluorophores (blue, green, red, and far-red) and phase out legacy tags such as GFP and mCherry. For example, mNeonGreen and mStayGold are brighter than GFP and are also fast-folding and photostable (Ko and Mizumoto 2025). For red fluorescence, mScarlet-I3 provides excellent performance and folding time (Cao et al. 2024), while new blue proteins such as mTagBFP2 or Electra2 (Papadaki et al. 2022) and far-red options such as miRFP680 or miRFP713 (Zhang et al. 2023b) allow straightforward multiplexing of up to 4 colors with commonly used microscopy filter sets. Beyond fluorophores, attaching an additional small epitope, such as 3xFLAG, would enable biochemical isolation of all tagged proteins (e.g. Dickinson et al. 2015). We suggest the ALFA tag should be tested further in *C. elegans*, as it displays higher affinity than FLAG (Götzke et al. 2019). The higher affinity also allows 1xALFA or 2xALFA configurations (Igreja et al. 2022), thus avoiding repetitive sequences that can cause problems during construct assembly. While covering routine biochemistry applications, ALFA also allows nanobody-based relocalization (Xu et al. 2022), degradation (Liu et al. 2024; Yang et al. 2025), and fluorescence or super-resolution imaging (Westlund et al. 2023; Quintin et al. 2025).

We propose WormTagDB as a coordination hub to register planned and newly generated alleles. This will allow researchers to avoid duplicate tagging and facilitate strain sharing and tagging designs. Each entry captures the essential metadata (gene, tag position, fluorophore, etc.). Crucially, negative results, such as failed tag positions and problematic linkers, can also be recorded. This registry will improve project planning and make strain sharing routine. WormTagDB can also be used by Undergraduate Research Experience courses (CUREs) for tagging projects (Hastie et al. 2019; McDonald et al. 2023; Herrera Sandoval et al. 2024).

As a centralized registry, WormTagDB initiates an organized path to tag the *C. elegans* proteome. As tagged allele inventories continue to grow, large-scale imaging pipelines will be able to process whole-worm datasets using automated segmentation, registration, and representation learning techniques to extract organ- and cell-level localization and dynamics. Proteome-scale tagging projects in yeast (Huh et al. 2003) and human cell lines (Cho et al. 2022) have demonstrated that such large-scale maps of protein localization and interactions are achievable and can be integrated with single-cell expression and perturbation data into a functional protein atlas. Because the worm is transparent and genetically tractable, *C. elegans* was the first multicellular animal to have its entire developmental cell lineage mapped and its genome fully sequenced (Corsi et al. 2015). Now, a comprehensive resource of endogenously tagged alleles to reveal the localization and functions of the entire proteome is within reach.

## Data Availability

All data necessary for confirming the conclusions presented in the article are represented fully within the article figures and supplementary information. Supplementary figures and tables can be found on figshare (https://doi.org/10.25387/g3.31743685). The data is viewable at the WormTagDB website (https://wormtagdb.rc.duke.edu), and all relevant code is available on GitHub (https://github.com/jakeleyhr/WormTagDB and https://github.com/jakeleyhr/CRISPR-Guide-and-Primer-Design-Pipeline-for-C.-elegans).
